# Children with COVID-19 behaving milder may challenge the public policies: a systematic review and meta-analysis

**DOI:** 10.1186/s12887-020-02316-1

**Published:** 2020-09-01

**Authors:** Chan Liu, Yu He, Lian Liu, Fang Li, Yuan Shi

**Affiliations:** Department of Neonatology, Children’s Hospital of Chongqing Medical University; National Clinical Research Center for Child Health and Disorders; Ministry of Education Key Laboratory of Child Development and Disorders, China International Science and Technology Cooperation base of Child Development and Critical Disorders; Chongqing Key Laboratory of Pediatrics, Chongqing, 400014 People’s Republic of China

**Keywords:** COVI-19, SARS-CoV-2, Children, Meta-analysis, School closure

## Abstract

**Background:**

The emerging virus is rampaging globally. A growing number of pediatric infected cases have been reported. Great efforts are needed to cut down the transmission.

**Methods:**

A single-arm meta-analysis was conducted. We searched PubMed, Google Scholar, Web of Science, and several Chinese databases for studies presenting characteristics of children confirmed with Coronavirus Disease 2019 (COVID-19) from December 12, 2019 to May 10, 2020. Quality Appraisal of Case Series Studies Checklist was used to assess quality and publication bias was analyzed by Egger’s test. Random-effect model was used to calculate the pooled incidence rate (IR) or mean difference (MD) with 95% confidence intervals (CI), or a fixed model instead when I^2^ < 50%. We conducted subgroup analysis according to geographic region. Additionally, we searched United Nations Educational Scientific and Cultural Organization to see how different countries act to the education disruption in COVID-19.

**Results:**

29 studies with 4300 pediatric patients were included. The mean age was 7.04 (95% CI: 5.06–9.08) years old. 18.9% of children were asymptomatic (95% CI: 0.121–0.266), 37.4% (95% CI: 0.280–0.474) had no radiographic abnormalities. Besides, a proportion of 0.1% patients were admitted to intensive care units (0, 95% CI: 0.000–0.013) and four deaths were reported (0, 95% CI: 0.000–0.000). Up to 159 countries have implemented nationwide school closures, affecting over 70% of the world’s students.

**Conclusion:**

Children were also susceptible to SARS-CoV-2, while critical cases or deaths were rare. Characterized by mild presentation, the dilemma that children may become a potential spreader in the pandemic, while strict managements like prolonged school closures, may undermine their well-beings. Thus, the public policies are facing challenge.

## Background

In December 2019, dozens of pneumonia cases with unknown etiology were reported in Wuhan, Hubei Province of China. Further sequencing analysis on samples of bronchoalveolar lavage fluid from pneumonia patients indicated that a new type of coronavirus, 2019 novel coronavirus (2019-nCoV), later renamed as severe acute respiratory syndrome coronavirus 2 (SARS-CoV-2), was to blame for this outbreak [1, 2]. The emerging disease caused by this pathogen, was then named Coronavirus Disease 2019 (COVID-19) officially by the World Health Organization (WHO). Human-to-human transmission has been recognized early onset of the spread of COVID-19 [3], and the numbers of confirmed cases keeps surging over the past few months. On 11 Mar 2020, the outbreak of COVID-19 was formally classified as a worldwide pandemic. As of 17 May, altogether 4,525,497 confirmed cases and 307,395 deaths across 215 countries were reported by WHO [4].

Though the SARS-CoV-2 is, based on current updated knowledge, phylogenetically, different from severe acute respiratory syndrome coronavirus (SARS-CoV) and Middle East Respiratory Syndrome-coronavirus (MERS CoV), which were identified as the cause of the two previous epidemics occurred in China and Saudi Arabia, they do share certain similarities. SARS-CoV-2 shares 79% genome sequence similarity to SARS-CoV and 50% genome sequence homology to MERS-CoV [5]. All the three viruses belong to Beta coronavirus and are enveloped positive-strand RNA viruses, patients got infected mainly manifested with respiratory symptoms (e.g. fever and cough) and poor clinical outcomes often associated with older age and underlying diseases [5, 6]. Children with SARS or MERS appeared to develop a milder clinical course, thus resulted in a significant low mortality in the two previous outbreaks [7, 8]. An earlier study on 2143 pediatric patients by Dong [9] and colleagues found that 3% of laboratory-confirmed cases were severe/critical, while 7.4% in suspected cases.

So far, SARS-CoV-2 infection has aroused grave concern globally, however, it seems that children got less focused due to a milder presentation. Evidence-based data is in an urgent need to make up the gap in understanding clinical spectrum of COVID-19 in children. Therefore, we are going to synthesize and summarize the clinical characteristics and epidemiology of children with COVD-19 based on the latest literatures to provide a systematic view towards pediatric patients.

## Methods

The protocol of this review followed recommendations established by Preferred Reporting Items for Systematic Reviews and Meta-Analyses (PRISMA) guidelines [10] and was registered in the International Prospective Register of Systematic Reviews (PROSPERO) database (ID: CRD42020173233).

### Search strategy

A systematic search was conducted in the following electronic databases: PubMed/Medline, Google Scholar, Web of Science, China National Knowledge Infrastructure (CNKI), Wanfang and several Chinese medical journals from December 1, 2019 to May 10,2020, incorporating the terms “COVID-19”, “SARS-CoV-2”, “children”, “pediatric” etc. No language limitations were applied. The detailed search strategy can be found in Additional file 1.

Additionally, we searched United Nations Educational Scientific and Cultural Organization (UNESCO, https://zh.unesco.org/) to find out how different countries act to the education disruption in COVID-19.

### Study selection

The studies included in this meta-analysis should meet the following criteria: (1) all types of studies either retrospective or prospective (e.g. cohort, cross-sectional study, case report, case series); (2) studies reporting information regarding COVID-19; (3) studies describing clinical characteristics of pediatric patients (0–19 years) diagnosed by RT-PCR; (4) clinical data of more than five cases can be drawn from the articles. Duplicate studies were removed. Studies that select cases from the same hospital during the same period were excluded to avoid regional bias and potential redundant report, then articles with maximum cases were retained. We also excluded studies that reported data on both adults and children, where we failed to extract pediatric data.

### Data extraction

Data were extracted from included studies by two reviewers (CL and LL) using Microsoft Excel 2019 independently, any disagreements were resolved by discussion with a third investigator (YH). We extracted study characteristics including study design, time of enrollment, institutions, sample size, study subject features age, gender, epidemiology, symptoms and signs(e.g., fever, cough, lack of symptom), laboratory findings (e.g. white blood cell counts [WBC], lymphocyte counts [L],et.), radiographic images, treatments and outcomes(e.g. discharged, death). Primary presentation described in each study were extracted with no assumptions.

### Assessment of methodological quality

Quality assessment of eligible studies was performed by the Quality Appraisal of Case Series Studies Checklist of the Institute of Health Economics (IHE) [11], which is comprised of 20 items. Each item would be scored ‘0’ if it was answered ‘NO’ or ‘UNCLEAR’, if the answer was ‘YES’, the item scored ‘1’. A study with 14 or more scores (≥ 70%) was considered to be of acceptable quality.

### Statistical analysis

The statistical software R 3.6.3 (R Foundation) was used to carry out the single-arm meta-analysis. Original data extracted from the literature will be transformed by the double arcsine method if the data is not normally distributed. Pooled incidence rates (IR) and 95% confidence intervals (95% CI) were calculated or dichotomous data and mean difference (MD) with 95% CI were used to report continuous data. The χ^2^ test and the I^2^ statistic were used to assess heterogeneity among studies with the random-effect model and DerSimonian and Laird method, or a fixed model instead when I^2^ < 50% (I^2^ > 50% indicated that heterogeneity was statistically significant). We also conducted a subgroup analysis according to geographic region (Wuhan and outside Wuhan) to explore reasons for heterogeneity. In addition, a sensitivity analysis was followed by.

Publication bias was assessed using funnel plots and Egger’s regression asymmetry test for meta-analysis that included at least 10 studies. *P*-value of < 0.05 indicated the existence of publication bias.

## Results

### Study search and characteristics

**Fig. 1:**
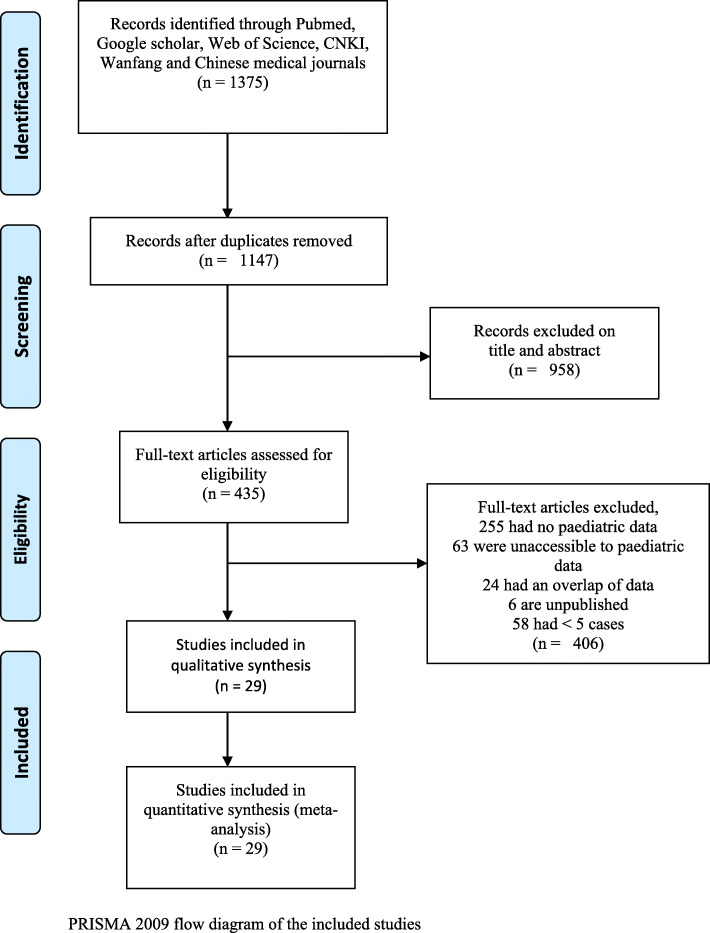
PRISMA 2009 flow diagram of the included studies

A total of 1375 relevant papers were identified after a systematic search. (see Fig. 1). For those which were accessible to pediatric data, we conducted a comprehensive screening and comparison according to time of enrollment, institutions and demographic characteristics of subjects, 24 articles were under suspicion of an overlapped data were removed. Of 29 studies [12–40] incorporating 4300 children included in this meta-analysis, 20 were case series, 4 cross-sectional, 3 prospective cohorts and 2 retrospective cohort, none compared cases with controls. Study size ranged from 5 to 2572 participants from six countries (China, Italy, United States, Canada, Spain, Rome). The detailed characteristics can be found in Supplementary Table 1 (see Additional file 2).

As of 17 May 2020, 159 countries were reported to have implemented nationwide school closures to mitigate the impact of COVID-19 on children, affecting over 70% of the world’s student population.

### Demographical characteristics and epidemiology

The mean age of pediatric patients enrolled in the 29 studies was 7.04 years old (95% CI: 5.06–9.08), range from 1 day to 19 years old. Particularly, 12% (95% CI:0.063–0.188) of children were less than 1 year old, 14.9% (95% CI:0.105–0.196) were 1 to 4 years old, 23.2% were 5 to 9 years, 23.1% were 10 to 14 years and 5.8% were more than 15 years old. Among them boys accounted for 53.6% (95% CI: 0.494-0.577). Comorbidities were reported in six studies with a proportion of 9.9% (95% CI: 0.002–0.215).

A large number of cases were identified as part of family clusters with COVID-19, the pooled incidence rate was up to 81.5% (95% CI:0.710–0.903). Besides, the pooled prevalence of cases associated with original epidemic area was 39.8% (95% CI:0.180–0.635).

### Severity of disease

Fourteen studies described the severity of COVID-19 in pediatric group with 1 patient diagnosed as critical type (0, 95% CI:0.000–0.006) and 2 as severe type (0, 95% CI: 0.000–0.006).

### Clinical manifestations

After a systematic review, we found 26 symptoms and signs reported in children infected with SARS-CoV-2. For the features of “sneezing”, “swollen tonsils”, “headache”, “wheeze”, “chill/rigor”, “chest pain/distress”, “abdominal pain”, “seizure/convulsion”, “rash”, “constipation”, “anosmia”, “arthralgia”, “conjunctivitis”, “cyonosis” and “tachycardia”, meta-analysis was thought to be unnecessary since few researches have presented. As is shown in Table 1, fever (52.7, 95% CI: 0.443–0.610) and cough (41.9, 95% CI:0.357–0.481) were the most prevalent and mild or moderate fever was more frequent than high fever. Lack of symptoms was also relatively common in these included cases, which turned out a proportion of 18.9% (95% CI: 0.121–0.266). Conversely, other symptoms or signs didn’t have such a frequent presentation (summarized in Table 1).

**Table 1:** Results of Meta-analysis on children with COVID-19

Characteristics	Events/Total	N of studies	Mean/Pooled incidence (%)	95% CI	I^**2**^ (%)	***P-*** value	Publication bias (***p***- value)
**Demographic information**							
Male	2305/4218	29	53.6	49.4–57.7	50	< 0.01	0.2161
Age (years)	4300/4300	29	7.04	5.06–9.08	99.79	< 0.01	< 0.05
< 1	602/4176	24	12.0	6.3–18.8	92	< 0.01	0.7211
1–4	456/3791	21	14.9	10.5–19.6	60	< 0.01	< 0.05
5–9	619/3791	21	23.2	17.7–29.0	67	< 0.01	< 0.05
10–14	960/3791	21	23.1	21.6–24.6	42	0.02	0.2341
15–19	1253/3962	21	5.8	0.9–13.3	95	< 0.01	< 0.05
Comorbidities	159/927	12	9.9	2.0–21.5	94	< 0.01	0.5658
**Epidemiology**							
Linkage to Wuhan	275/465	21	39.8	18.0–63.5	95	< 0.01	< 0.05
Family cluster	525/704	20	81.5	71.0–90.3	86	< 0.01	0.5347
**Severity of disease**							
Mild & Common	344/347	14	100.0	99.1–100.0	0	1.00	0.4795
Severe	2/347	14	0	0.0–0.6	0	1.0	0.0968
Critical	1/347	14	0	0.0–0.5	0	1.0	< 0.05
**Clinical manifestations**							
Asymptomatic	248/1726	28	18.9	12.1–26.6	86	< 0.01	< 0.05
Fever	941/2017	29	52.7	44.3–62.0	87	< 0.01	0.1689
Mild (37.7 °C–38.0 °C)	72/426	14	19.2	12.0–27.4	56	< 0.01	0.0575
Moderate (38.1 °C–39.0 °C)	95/433	15	15.5	9.0–22.9	54	< 0.01	0.0819
High (39.1 °C-)	44/438	15	8.2	2.8–15.3	66	< 0.01	0.5681
Cough	1035/2017	29	41.9	35.7–48.1	72	< 0.01	< 0.05
Expectoration	14/270	17	1.4	0.0–4.1	43	0.03	0.464
Pharyngeal erythema	105/429	17	6.0	0.0–19.1	91	< 0.01	< 0.05
Sore throat	425/1985	27	5.0	0.6–11.8	93	< 0.01	< 0.05
Rhinorrhea	455/1827	27	3.5	0.1–9.8	93	< 0.01	< 0.05
Stuffy nose	26/592	24	1.0	0.1–2.5	46	< 0.01	0.4097
Diarrhea	98/1021	26	4.2	1.8–7.3	45	< 0.01	< 0.05
Vomiting	69/1021	26	3.5	2.1–5.1	35	0.04	0.0632
Tachypnea/dyspnea	117/1057	27	2.5	1.6–4.8	72	< 0.01	< 0.05
Fatigue/myalgia	103/1044	25	2.7	0.3–6.4	73	< 0.01	< 0.05
**Laboratory findings**							
WBC decreased	86/509	20	10.6	5.4–16.8	60	< 0.01	0.1130
WBC increased	38/302	18	10.3	6.6–14.6	0	0.62	0.6663
L decreased	56/497	19	10.8	3.9–19.7	80	< 0.01	0.131
L increased	33/182	13	15.4	9.8–21.7	43	0.05	0.7734
ALT increased	39/405	15	6.5	3.8–9.6	43	0.04	0.3616
AST increased	58/423	14	10.9	5.0–18.2	65	< 0.01	0.7864
LDH increased	51/183	13	23.0	8.8–38.3	79	< 0.01	0.7704
CRP increased	107/537	18	12.3	5.4–21.0	77	< 0.01	0.2760
**Radiographic evaluation**							
Normal	166/501	23	37.4	28.0–47.4	77	< 0.01	< 0.05
GGO	169/456	19	35.7	31.0–40.5	49	< 0.01	0.6935
Consolidation	38/224	14	10.5	1.6–23.6	80	< 0.01	0.7874
Unilateral compromised	93/365	15	28.2	19.4–37.8	55	0.01	0.0912
Bilateral compromised	74/365	15	21.9	10.4–35.5	80	< 0.01	0.2329
**Therapy**							
Oxygen therapy	38/480	17	4.9	2.7–7.5	22	0.2	0.2031
Antiviral treatmen							
Interferon	140/370	15	63.0	25.5–93.9	98	< 0.01	< 0.05
Lopinavir-ritonavir	68/216	13	26.0	11.8–42.7	83	< 0.01	0.5968
Ribavirin	14/216	13	2.9	0.5–6.4	46	0.03	0.8658
Oseltamivir	40/216	13	10.5	0.5–27.2	88	< 0.01	0.755
Arbidor	35/216	13	5.9	0.0–17.8	82	< 0.01	0.1585
Antibiotics	23/182	13	11.3	1.8–25.4	79	< 0.01	0.1121
Corticosteroid	6/387	16	0.0	0.0–0.4	13	0.30	< 0.05
Immunoglobin	8/381	16	0.0	0.0–0.3	55	< 0.01	< 0.05
Mechanic ventilation	6/737	23	0.0	0.0–0.2	0	1.0	0.1143
**Outcomes**							
Discharged	419/553	20	84.1	69.6–95.1	92	< 0.01	0.7479
ICU admission	32/1587	28	0.1	0.0–1.3	51	< 0.01	0.1641
Death	4/4278	14	0.0	0.0–0.0	0	1	< 0.05

Note: *WBC* white blood cell counts, *L* lymphocyte counts, *AST* aspartate aminotransferase, *ALT* alanine aminotransferase, *LDH* lactate dehydrogenase, *CRP* C-reactive protein, *GGO* ground-glass opacity, *ICU* intensive care unit

If the observed index wasn’t reported in a research, 0 cases were calculated as occurred

Linkage to Wuhan referred to children who resided in Wuhan or travelled to Wuhan or contacted with people from Wuhan before the onset of infection

Family cluster was defined as more than one infected family member residing with the child

### Laboratory findings

The frequency of decreased WBC was similar to increased WBC in reported cases, the pooled incidence rate was 10.6% (95% CI: 0.054–0.168) and 10.3% (95% CI: 0.066–0.146). Compared to lymphopenia (10.8, 95% CI: 0.039–0.197), the incidence of lymphocytosis (15.4, 95% CI: 0.098–0.217) was slightly higher in pediatric patients. Increased C-Reactive Protein (CRP) was in 12.3% (95% CI: 0.054–0.210) of subjects. The pooled incidence rate of an elevated level of aspartate aminotransferase (AST), alanine aminotransferase (ALT) and lactate dehydrogenase (LDH) were 10.9% (95% CI:0.050–0.182), 6.5% (95% CI:0.038–0.096), 23.0% (95% CI:0.088–0.383) respectively.

### Radiographic findings

Normal radiologic presentation was reported in 37.4% (95% CI: 0.280–0.474) of cases. Apart from that, the most common manifestation was ground-glass opacity (GGO) (35.7, 95% CI:0.310–0.405), unilateral compromised lesions were more frequently presented than bilateral (28.2, 95% CI:0.194–0.378 vs 21.9,95% CI: 0.104–0.355). Additionally, 10.5% of subjects (95% CI:0.016–0.236) were reported with consolidation on computed tomography (CT) imaging.

### Treatments and clinical outcomes

Approximate 63.0% (95% CI: 0.255–0.939) SARS-CoV-2 infected children were treated with interferon and lopinavir-ritonavir (26, 95% CI: 0.255–0.939) was more frequently applied compared with other antiviral agents including ribavirin, oseltamivir and arbidor. Few cases received administration of corticosteroid, immunoglobin therapy and mechanic ventilation, the pooled incidence rate was 0.0% (95% CI: 0.0000–0.004), 0.0% (95% CI: 0.000–0.003) and 0.0%(95% CI:0.000–0.002), respectively.

The majority of patients (84.1%,95% CI: 0.696–0.951) got discharged from hospital and 0.1% (95% CI: 0.000–0.013) were transferred to intensive care units. Unfortunately, 4 deaths (0,95% CI: 0.000–0.000) were ultimately confirmed.

### Subgroup analysis and sensitivity analysis

The results of heterogeneity assessment and publication bias are shown in Table 1. Subgroup analysis indicated that geographic region may account for the heterogeneity of “fever (mild)” and “unilateral compromised”. (Table 2). A further exploration for between-study heterogeneity by sensitivity analysis showed that none of these studies should be excluded.

**Table 2:** Subgroup analysis on the characteristics of children with COVID-19

Characteristics	Wuhan			Outside Wuhan		
	R (95% CI)	I2	***p***-value	R (95% CI)	I2	***p***-value
**Demographic information**						
Male	43.5(17.3–71.6)	69%	0.04	54.8(53.2–56.5)	49%	< 0.01
Age						
< 1y	38.7(0.0–96.5)	94%	< 0.01	9.4(4.1–16.1)	91%	< 0.01
1-4y	31.8(0.0–1.0)	93%	< 0.01	8.8(7.7–9.9)	48%	0.01
5-9y	3.5(0.0–22.8)	35%	0.22	24.5(18.8–30.5)	69%	< 0.01
10-14y	0.0(0.0–12.4)	0%	0.90	23.3(21.8–24.8)	31%	0.09
15-19y	0.0(0.0–12.4)	0%	0.00	8.7(3.3–15.7)	90%	< 0.01
Comorbidities	0.0(0.0–1.6)	0%	0.80	12.6(3.5–25.4)	92%	< 0.01
**Severity of illness**						
Mild & Common	–	NA	NA	1.0(99.1–1.0)	0%	0.99
Severe	–	NA	NA	0.0(0.0–0.6)	0%	1.00
Critical	–	NA	NA	0.0(0.0–0.5)	0%	1.00
**Epidemiology**						
Linkage to Wuhan	96.4(70.4–1.0)	78%	0.01	29.7 (21.0–39.0)	54%	< 0.01
Family cluster	92.5(87.6–0.965)	0%	0.33	79.2(68.0–88.8)	83%	< 0.01
**Clinical manifestations**						
Asymptomatic	19.1(13.0–25.9)	17%	0.30	19.9(12.2–28.6)	86%	< 0.01
Fever	67.0(25.9–97.8)	83%	< 0.01	51.6(42.5–60.7)	87%	< 0.01
Mild (37.7 °C–38.0 °C)	6.2(2.4–11.2)	0%	0.54	20.8(15.5–26.5)	23%	0.21
Moderate (38.1 °C–39.0 °C)	19.1(13.0–25.9)	17%	0.30	15.1(7.0–24.9)	60%	< 0.01
High (39.1 °C-)	54.7(0.0–1.0)	96%	< 0.01	5.9(2.7–9.8)	0%	0.48
Cough	61.5(23.2–93.7)	81%	< 0.01	40.2(33.7–46.8)	73%	< 0.01
Expectoration	14.3(0.0–51.7)	NA	NA	1.3(0.0–3.9)	44%	0.03
Pharyngeal erythema	38.1(3.9–80.0)	84%	< 0.01	2.3(0.0–10.7)	80%	< 0.01
Sore throat	0.0(0.0–0.0)	0%	0.56	6.2 (1.4–13.0)	91%	< 0.01
Rhinorrhea	4.9(1.5–9.5)	0%	0.45	3.8 (0.0–11.6)	93%	< 0.01
Stuffy nose	1.8(0.0–5.4)	0%	0.94	1.6(0.0–5.1)	51%	< 0.01
Diarrhea	4.8(1.4–9.5)	0%	0.70	4.1(1.4–7.7)	51%	< 0.01
Vomiting	14.4(0–52.9)	83%	< 0.01	3.4(1.9–5.1)	18%	0.22
Tachypnea/dyspnea	16.4(1.6–38.6)	55%	0.11	4.3(2.7–6.1)	47%	< 0.01
Fatigue/myalgia	4.5(1.3–9.1)	0%	0.65	2.6(0.1–6.8)	75%	< 0.01
**Laboratory findings**						
WBC decreased	39.6(6.6–78.4)	74%	0.05	8.4(5.2–12.2)	26%	0.15
WBC increased	0.0(0.0–26.8)	NA	NA	10.7(6.9–15.1)	0%	0.62
L decreased	48.4(0.0–1.0)	97%	< 0.01	8.8(3.0–16.5)	68%	< 0.01
L increased	0.0(0.0–26.8)	NA	NA	16.3(10.5–22.8)	43%	0.06
ALT increased	9.9(5.2–15.5)	0%	0.57	4.2(1.4–8.0)	41%	0.06
AST increased	33.4(0.0–86.0)	86%	< 0.01	8.9(3.1–16.7)	60%	< 0.01
LDH increased	33.3(1.3–76.4)	NA	NA	21.4(7.8–38.5)	80%	< 0.01
CRP increased	45.9(0.0–98.9)	90%	< 0.01	9.6(2.7–19.0)	76%	< 0.01
**Radiographic evaluation**						
Normal	13.1(7.7–19.3)	0%	0.62	39.7(29.8–50.1)	66%	< 0.01
GGO	30.9(23.6–38.6)	0%	0.67	38.1(29.1–47.5)	51%	< 0.01
Consolidation	0.0(0.0–31.7)	NA	NA	11.4(1.9–25.3)	82%	< 0.01
Unilateral compromised	13.6(3.6–27.3)	18%	0.27	30.6(23.6–37.9)	40%	0.07
Bilateral compromised	38.0(0.0–98.7)	90%	< 0.01	20.3(7.7–36.0)	78%	< 0.01
**Therapy**						
Antiviral treatment						
Interferon	100.0(73.2–100.0)	NA	NA	59.5(21.5–92.5)	94%	< 0.01
Lopinavir-ritonavir	0.0(0.0–26.8)	NA	NA	28.5(13.6–45.8)	83%	< 0.01
Ribavirin	33.3(1.3–76.4)	NA	NA	2.6(0.4–6.0)	39%	0.08
Oseltamivir	100.0(73.2–100.0)	NA	NA	6.1(0.0–19.2)	85%	< 0.01
Arbidor	0.0(0.0–26.8)	NA	NA	6.5(0.0–19.3)	84%	< 0.01
Antibiotics	100.0(73.2–100.0)	NA	NA	6.5(0.8–15.2)	61%	< 0.01
Corticosteroid	66.7(23.6–98.7)	NA	NA	0.0(0.0–0.2)	0%	1.00
Immunoglobin	16.7(0.0,58.6)	NA	NA	0.5(0.0–3.8)	54%	< 0.01
Mechanic ventilation	0.0(0.0–1.4)	0%	0.96	0.0(0.0–0.2)	0%	1.00
**Outcome**						
Discharged	90.3(84.7–94.9)	0%	0.38	82.6(64.4–95.9)	92%	< 0.01
ICU admission	0.5(0.0–7.5)	28%	0.25	0.1(0.0–1.4)	53%	< 0.01
Death	0.0 (0.0–0.2)	0	0.81	0.0 (0.0–0.0)	0%	0.97

**Note:** *WBC* white blood cell counts, *L* lymphocyte counts, *AST* aspartate aminotransferase, *ALT* alanine aminotransferase, *LDH* lactate dehydrogenase, *CRP* C-reactive protein, *GGO* ground-glass opacity, *ICU* intensive care unit

NA: not applicable, only one or no study included in the subgroup

## Discussion

The unpredictable emergency of SARS-CoV-2 has posed a substantial threat to public health. Implementing efforts on aggregating the existing data about epidemiology, clinical, laboratory, and imaging characteristics to have a better understanding of the virus, its patterns of spread and the spectrum of illness is of critical significance. Through a comprehensive searching, a total of 29 articles with 4300 cases were included.

The proportion of male to female of this analysis (53.6% vs 46.4%) is similar to the gender distribution in an initial investigation [9](57.5% vs 42.5% in 731 confirmed cases) and general population [41] (55.9% vs 44.1%) (Table 3). All the results seem to show that male have a slightly higher incidence than female in COVID-19.

**Table 3:** Comparison of incidence of clinical characteristics between children with COVID-19, general population with COVID-19, children with SARS and children with H1N1 influenza

	Children with COVID-19	General population with COVID-19 [41]	Children with SARS [42]	Children with H1N1 influenza [43]
Age(y-old)	5.5(3.44–7.65)	51.97 (46.06–57.89)	12.2	5
Male	53.6% (49.4–57.7)	55.9% (51.6–60.1)	45.5%	54.7%
Asymptomatic	18.9% (12.1–26.6)	–	0	< 6.1%
Fever	52.7% (44.3–61.0)	88.7% (84.5–92.9)	100%	93.9%
Cough	41.9% (35.7–48.1)	57.6% (40.8–74.4)	63.6%	88.5%
Sore throat	5.0% (0.6–11.8)	11.0% (2.8–19.2)	13.6%	19.6%
Diarrhea	4.2% (1.8–7.3)	6.1% (2.4–9.7)	20.5%	6.1%
Tachypnea/Dyspnea	2.5% (1.6–4.8)	45.6% (10.9–80.4)	9.1%	–
Leucopenia	10.0% (4.5–16.7)	18.7% (8.5–28.8)	34.1%	16.9%
Lymphopenia	10.8% (3.9–19.7)	43.1% (18.9–67.3)	77.3%	34.5%
Ground-glass opacity	35.7% (31.0–40.5)	68.5% (51.8–85.2)	–	–
**Comorbidities**	3% (11/361)	36.8% (24.7–48.9)	11.4%	14.9%
ICU admission	0.1% (0.0–1.3)	20.3% (10.0–30.6%)	11.4%	19.6%
Death	0 (0.0–0.0)	13.9% (6.2–21.5)	0	2%
N	4300	2874	44	148

Note: The results of characteristics of COVID-19 in children and general population were presented with pooled incidence and 95% CI, characteristics of “comorbidities” in children with COVID-19 were presented with incidence(n/N) due to insufficient data

No meta-analysis results of characteristics of children with SARS and H1N1 influenza were found, incidence(n/N) was presented as a substitute

“-”: not available

According to our results, children got infected with SARS-CoV-2 mainly through family clustering, quite the same as SARS-CoV [7] and MERS-CoV [8].While compared to adults, children are more likely to be asymptomatic or present with milder symptoms, this reminds us that, whenever there’s a family member caught with this virus, it is necessary to conduct a virologic screening test on the child as soon as possible. Otherwise, the infected child may become a threat to other vulnerable populations (e.g. elderly people or people with severe underlying disease), resulting in further extension of ongoing pandemic, as was seen during influenza outbreak [44].

Fever and cough are the most common symptoms in COVID-19 children, in our study, the pooled incidence of fever is 52.7%, which is lower than that in adults [41], SARS [7, 42] and influenza [43]. Clearly, children with COVID-19 rarely had obvious signs and symptoms of upper respiratory tract (pharyngeal congestion, rhinorrhea, sore throat, stuffy nose). Through a comprehensive review, it’s easy to draw the conclusion that SARS-CoV-2 leads to a less aggressive clinical course in children with more asymptomatic and fewer symptoms, compared to that in adults and the other two pathogens. (Table 3).

In terms of laboratory abnormalities, only 10.8% of infected children presented with lymphopenia, which is quite different from findings in COVID-19 adults [41] and SARS [7].Besides, leucopenia was found in 10.6% of patients, nevertheless, a research including 80 virologic-confirmed children cited by Henry [45] reported 46% of lymphopenia. Theoretically, virus particles primarily spread through the respiratory mucosa, initially using the angiotensin-converting enzyme 2(ACE2) receptor (the cell-entry receptor for SARS-CoV-2) at ciliated bronchial epithelial cells and infect other cells, induce a cytokine storm in the body, generate a series of immune responses, and cause changes in peripheral white blood cells and immune cells such as lymphocytes [46, 47]. Presumptions have been made that children may be protected against SARS-CoV-2 because this enzyme is less mature at a younger age, since the immune system undergoes substantial changes from birth to adulthood. In general, WBC and lymphocyte remained normal in the majority of pediatric patients, suggesting that the newly emerging virus, SARS-CoV-2, may have a marginal influence on the immune function of children.

As for radiologic aspects, our research found that a proportion of 37.4% of 501 virologic positive cases were in absence of CT abnormalities, and Ground glass opacity, also typical signs of severe acute respiratory syndrome (SARS) [7], was shown in 35.7% of pediatric patients. This kind of low sensitivity hints us that routinely radiologic scans should not be overemphasized for screening or early identification of COVID-19 in children in consideration of substantial radiation exposure, especially when the child is lack of symptoms or running a mild clinical course. Therefore, more strict strategies and screening practices are required for the better management of pediatric cases.

Compared to adults [41], the spread of SARS-CoV-2 yield a much better prognosis in pediatric patients, similar to SARS [7]and Middle East Respiratory Syndrome (MERS) [8]. 84.1% of cases were discharged, the discharge rate ought to be higher actually since many children were still in hospital before the submission of the papers. The reasons why children experience a milder COVID-19 disease remain elusive. One possible explanation is that the response of children to SARS-CoV-2 is fundamentally different from that of adults, as demonstrated in earlier reports [48], the frequency of lymphopenia found in adults suggests that SARS-CoV-2 might act on lymphocytes, which is rare in children. Prior exposure to other respiratory virus may exert an influence, making children’s immune systems more resilient [7]. Besides, some researchers proposed that the mild disease in children may be associated with trained immunity, which refers to the use of certain vaccines such as Bacille de Calmette Guerin (BCG). BCG has been proved to provide nonspecific protection of mice against influenza virus infection probably by the induction of trained immunity [44]. In addition, the virulence and pathogenicity of the virus may decrease in pediatric patients who are usually belong to the second or third generation infection. Accordingly, further studies in fields of immunology, anatomy and virology are required to ravel out this puzzle.

With massive public health interventions implemented actively and effectively, the spread of SARS-CoV-2 seems to have been under control in several countries. On 17 May 2020, there were only 7 newly confirmed cases across mainland China and 4 were imported [49]. At present, while some countries are considering enhancing control measures, China is planning to lift restrictions, work resumes and school starts are on agenda. Nevertheless, concerns have been proposed that a second wave of cases might occur in light of the absence of herd immunity against COVID-19, escalating case importation or residual infected seeds and resumption of economic activities [50, 51]. It’s plausible to suggest whether children have to get away from school again to mitigate the revival transmission. School closures can affect the spread of virus during a pandemic through reducing transmission and new cases, while long periods of social distancing interventions in school may put students in a disadvantaged situation. Recently, some scholars are questioning the benefits brought by closing schools. On the one hand, school closures are based on empirical evidence and assumptions from influenza outbreaks, it’s hard to say such measures are also effective in coronavirus outbreaks like SARS, MERS and especially COVID-19, for which transmission dynamics appear to be different [52]. A systemic review [52] concluded that school closures in SARS did not contribute to the control of the epidemic and its effectiveness in COVID-19 would be less than other social distancing interventions, with only 2–4% of death prevention. Meanwhile, less comprehensive and deliberate plan can result in a completely converse consequence. Jude Bayham and Eli P Fenichel [53] estimated that school closures could lead to mortality rate increased by 0.35% and a greater number of deaths than they prevent when the health-care workforce declines by 15.0% due to unintended childcare obligations. (Table 4 shows alternative closure strategies in five countries). On the other hand, prolonged school dismissals can be detrimental to children’s physical and mental health [59, 60]. Out of school means a totally altered lifestyle—for example, fewer physical activities, less interaction with peer groups and longer screen time. Besides, many schools are offering online courses, but this is not available to all, especially to children from low socioeconomic households, and they may be further disadvantaged by nutrition shortfalls. Moreover, with home confinement, communities lockdown and economic recession deepens, family conflicts are rising, children are more likely to be exposed to domestic violence and abuse. Consequently, it is imperative for the policy makers to weigh the benefits of school closure against its costs carefully and deliberately and provide alternative strategies to minimize the adverse impacts of the COVID-19 on children’s well-being.

**Table 4:** School strategies in different countries in response to COVID-19

UK [54]	Localized closures have been implemented since 28 Feb. All educational settings are closed to everyone except the children of critical workers and vulnerable children^a^ since 20 March and will stay closed until further notice.
US [55]	School-based strategies (e.g., short-term or extended dismissals, event cancellations, social distancing measures) are adopted locally in collaboration with local health officials based on level of community transmission of COVID-19 and presence of COVID-19 cases within the school, combined with open child care programs^b^ like private child care centers for essential service providers. The majority of States have mandated school closures since 10 April, including until the end of the academic year in June. Some States, however, have recommended but not mandated the school closures.
Italy [56]	Some schools in the heaviest hit area have been shut down since 24 Feb. Mandatory closure of all schools and universities across the country were implemented from 10 March and will remain shut until 3 May.
France [57]	All nurseries, schools, colleges, high schools and universities are closed from 16 March and will gradually reopen from 11 May with the exception of universities, which will not reopen until the summer. Childcare services are established for staff who are essential to the management of the health crisis.
German [58]	Temporarily closing kindergartens and schools and postponing restart of colleges were implemented in state levels since mid-March are to be extended until 3 May 2020. Schools remain open for those who are willing to continue classes in some states. Daycare centres are available and will continue and will be extended to other occupational and needed groups.

Note: ^a^Vulnerable children include children who are supported by social care, those with safeguarding and welfare needs, including child in need plans, on child protection plans, ‘looked after’ children, young carers, disabled children and those with education, health and care (EHC) plans

^b^Other open child care programs are home-based child care, pre-kindergarten programs, Head Start and Early Head Start programs, temporary child care centers, and child care centers that partner with healthcare facilities to support healthcare workers who need child care

There are several limitations need to be acknowledged. Firstly, most of articles included in this meta-analysis are descriptive and retrospective with wide range of sample size, which highlighted different aspects of the illness, consequently, high heterogeneity was inevitable. Secondly, reports derived from China dominated the largest part, data from other countries are still in short. In addition, we have intended to conduct a subgroup analysis based on age stratification and severity of the disease, while enough information was unavailable. Therefore, the findings of this meta-analysis still need to be updated by more relevant studies with more strict design and larger sample size.

## Conclusions

In conclusion, our study highlights the epidemiology, clinical characteristics of COVID-19 in pediatric patients. This quantitative analysis provides evidence-based knowledge for the diagnosis and management in pediatric patients in the ongoing pandemic. Children were also susceptible to SARS-CoV-2. Compared to adults, children experienced a milder clinical course. The most frequent symptoms were fever and cough, asymptomatic were also quite common. Children with no or mild symptoms should be virologic-screened and isolated from immunocompromised populations at once when a family member is diagnosed with COVID-19 to prevent child-driven transmission. A group of children were absent from CT abnormalities, CT scans should not be overemphasized to avoid excessive radiation exposure. Public health officials should attach importance to additional childcare programs to protect the well-being of children in this pandemic context.

## Supplementary information

**Additional file 1.** SEARCH STRATEGY: This file describes the search strategy of this meta-analysis

**Additional file 2: Table S1.** Characteristics of the included studies

## Data Availability

Data sharing is not applicable to this article as no datasets were generated or analysed during the current study.

## Abbreviations

2019-nCoV: 2019 novel coronavirus
ACE2: Angiotensin-converting enzyme 2
ALT: Alanine aminotransferase
AST: Aspartate aminotransferase
BCG: Bacille de Calmette Guerin
COVID-19: Coronavirus Disease 2019
CRP: C-Reactive Protein
CT: Computed tomography
GGO: Ground-glass opacity
IHE: Institute of Health Economics
L: Lymphocyte counts
MERS: Middle East Respiratory Syndrome
MERS-CoV: Middle East Respiratory Syndrome-coronavirus
SARS: Severe acute respiratory syndrome
SARS-COV: Severe acute respiratory syndrome coronavirus
SARS-CoV-2: Severe acute respiratory syndrome coronavirus 2
WBC: White blood cell counts
WHO: World Health Organization
