# Supplementary material for: Children with COVID-19 behaving milder may challenge the public policies: a systematic review and meta-analysis
Source: BMC Pediatr. 2020 Sep 1;20:410. doi: 10.1186/s12887-020-02316-1 (PMC7459157; doi:10.1186/s12887-020-02316-1)
Supplement: Supplementary file 1 — Additional file 1. SEARCH STRATEGY: This file describes the search strategy of this meta-analysis [file 12887_2020_2316_MOESM1_ESM.pdf]

SEARCH STRATEGY

Chan Liu[1], Yu He[1], Lian Liu[1], Fang Li[1],Yuan Shi[1]

1: Department of Neonatology, Ministry of Education Key Laboratory of Child Development and Disorders; National Clinical Research Center for Child Health and Disorders; China International Science and Technology Cooperation base of Child development and Critical Disorders; Children's Hospital of Chongqing Medical University; Chongqing Key Laboratory of Pediatrics, Chongqing, 400014, P.R China. Fang Li: rematalili@hospital.cqmu.edu.cn,Yuan Shi: shiyuan@hospital.cqmu.edu.cn.

Online sources

- PubMed (http://www.ncbi.nlm.nih.gov/pubmed)
- Google Scholar (http://scholar.google.com/)
- Web of Science (https://webofknowledge.com/)
- CNKI(https://www.cnki.net/)
- Wanfang(http://www.wanfangdata.com.cn/index.html)
- Manual search
  - Chinese Journal of Pediatrics| Chin J Pediatrics
  - Chinese Journal of Contemporary Pediatrics | Chin J Contemp Pediatrics
  - Journal of Applied Clinical Pediatrics
  - Chinese Journal of Perinatal Medicine
  - Chinese Journal of Neonatology
  - Chinese Journal of Evidence Based Pediatrics | Chin J Evid Based Pediatrics

Boolean keywords/search terms

“COVID-19” OR” SARS-CoV-2” OR” COVID-2019” OR ”2019-nCOV” OR” 2019 novel coronavirus infection” OR” coronavirus disease-19” OR” coronavirus disease 2019” OR ” severe acute respiratory syndrome coronavirus 2” OR ” Novel Infected Coronavirus Pneumonia” OR ”Wuhan pneumonia” OR ”novel coronavirus”

“children” OR “infant” OR “neonate” OR” young” OR” newborn” OR” baby” OR” babies” OR” adolescent” OR” adolescence” OR “pediatric” OR” paediatric” or “juvenile” or “teenager”
