# Supplementary material for: Children with COVID-19 behaving milder may challenge the public policies: a systematic review and meta-analysis
Source: BMC Pediatr. 2020 Sep 1;20:410. doi: 10.1186/s12887-020-02316-1 (PMC7459157; doi:10.1186/s12887-020-02316-1)
Supplement: Supplementary file 2 — Additional file 2: Table S1. Characteristics of the included studies [file 12887_2020_2316_MOESM2_ESM.docx]

**Supplementary Table 1 Characteristics of the included studies.**

|  | **First Author** | **Time of Enrollment** | **Institution** | **Country** | **Study Type** | **N** | **Quality Assessment** | |
| --- | --- | --- | --- | --- | --- | --- | --- | --- |
|  |  |  |  |  |  |  | **Score** | **Result** |
| 1 | Yun Zhou^12^ | 1.20-2.01 | Shenzhen Third People's Hospital | China | Case series | 9 | 11 | Unacceptable |
| 2 | Weiyong Liu^13^ | 1.02-2.08 | Three branches of Tongji Hospital | China | Case series | 6 | 13 | Unacceptable |
| 3 | Duan Wang^14^ | 1.25-2.21 | Hospitals from six provinces in northern China^a^ | China | Case series | 31 | 14 | Acceptable |
| 4 | Zhiliang Hu^15^ | 1.16-2.19 | The Second Hospital of Nanjing | China | Cross-sectional | 6 | 15 | Acceptable |
| 5 | Yi Xu^16^ | 1.23-2.01 | Guangzhou Women and Children’s Medical Center | China | Prospective cohort study | 10 | 16 | Acceptable |
| 6 | Cai Jiehao^17^ | 1.19-2.03 | Children’s Hospital in six provinces^b^ | China | Case series | 10 | 15 | Acceptable |
| 7 | Xiaoxia Lu^18^ | 1.28-2.26 | Wuhan Children’s Hospital | China | Case series | 171 | 17 | Acceptable |
| 8 | Haiyan Qiu^19^ | 1.17-3.01 | Three hospitals in Zhejiang^c^ | China | Retrospective cohort study | 36 | 17 | Unacceptable |
| 9 | Wei Li^20^ | 1.28-2.08 | The Fifth Affiliated Hospital of Sun Yat-sen University | China | Prospective cohort study | 5 | 13 | Unacceptable |
| 10 | Kai Feng^21^ | 1.16-2.06 | Shenzhen Third People's Hospital | China | Case series | 15 | 12 | Unacceptable |
| 11 | Min Wei^22^ | 12.08-2.06 | Renmin Hospital of Wuhan University | China | Case series | 9 | 16 | Acceptable |
| 12 | Zhong Zheng^23^ | 1.10-2.25 | Radiology Quality Control Center in Hunan | China | Case series | 9 | 13 | Unacceptable |
| 13 | Xiang Ma^24^ | 1.25-2.06 | Qilu Children’s Hospital of Shandong University | China | Case series | 6 | 13 | Unacceptable |
| 14 | TAN Xin^25^ | 1.17-2.29 | First Hospital of Changsha | China | Case series | 13 | 12 | Unacceptable |
| 15 | Li Zhu^26^ | 1.24-2.22 | Three designated hospitals in three cities of Jiangsu | China | Case series | 10 | 12 | Unacceptable |
| 16 | Yu-pin Tan^27^ | 1.27-3.01 | The First Affiliated Hospital of Hunan Normal University | China | Case series | 10 | 13 | Unacceptable |
| 17 | Juan Chen^28^ | 1.28-2.11 | YongChuan hospital of ChongQing medical university, Chongqing Three Gorges Central Hospital, The Public Health Center | China | Case series | 12 | 13 | Unacceptable |
| 18 | Rui Song^29^ | 1.16-1.29 | Beijing Ditan Hospital | China | Cross-sectional | 7 | 15 | Acceptable |
| 19 | Wenliang Song^30^ | 1.31-3.17 | Central Hospital of Xiangyang | China | Case series | 16 | 16 | Acceptable |
| 20 | WU Hua-Ping^31^ | 1.21-2.29 | Designated hospitals in Jiangxi | China | Case series | 23 | 15 | Acceptable |
| 21 | Niccolò Parri^32^ | 3.03-3.27 | 17 pediatric emergency departments | Italy | Retrospective cohort study | 100 | 17 | Acceptable |
| 22 | Anna Maria Musolino^33^ | 3.15- | Two tertiary pediatric hospitals | Rome | Prospective cohort study | 10 | 16 | Acceptable |
| 23 | Bin Zhang^34^ | 1.20-3.09 | Four tertiary-care hospitals in Guangdong, Hunan and Hubei^d^ | China | Case series | 46 | 14 | Acceptable |
| 24 | Silvia Garazzino^35^ | 3.25-4.01 | 11 paediatric hospitals and 51 paediatric units | Italy | Cross-sectional | 168 | 17 | Acceptable |
| 25 | Elena Cela^36^ | NA | NA | Spain | Cross-sectional | 15 | 15 | Acceptable |
| 26 | Dana Paquette^37^ | 1.15-4.27 | Public Health Agency of Canada | Canada | Case series | 938 | 14 | Acceptable |
| 27 | CDC COVID-19 Response Team^38^ | 2.12-4.02 | Centers for Disease Control and Prevention | United States | Case series | 2572 | 14 | Acceptable |
| 28 | Bo Li^39^ | 1.16-3.14 | Yichang Central People’s Hospital | China | Case series | 22 | 12 | Unacceptable |
| 29 | Li Yunjiang^40^ | 1.24-2.10 | Hangzhou Xixi Hospital | China | Case series | 15 | 13 | Unacceptable |

Note: Quality assessment based on 20 items of Quality Appraisal of Case Series Studies Checklist of the IHE: 14 or more scores (≥ 70%) considered to be acceptable.

NA: not available

a: 21 hospitals in 17 cities of six provinces of Shaanxi, Gansu, Ningxia, Hebei, Henan and Shandong: XI′an Children′s Hospital, Qilu Hospital of Shandong University, General Hospital of Ningxia University, Fourth People′s Hospital of Ningxia Hui Autonomous Region, Xi′an eighth hospital, Ankang Central Hospital, Tongchuan Mining Bureau Central Hospital, Yan′an Second People′s Hospital Affiliated to Yan′an University, Lanzhou Pulmonology Hospital, Gansu Provincial Maternity and Child‑Care Hospital, Xianyang Central Hospital, Children′s Hospital of Zhengzhou University, Zhumadian Center Hospital, Shijiazhuang Fifth Hospital, the Affiliated Children′s Hospital of Xi′an Jiaotong University, Tengzhou Central People′s Hospital, Heze Municipal Hospital, People′s Hospital of Rizhao, Yantai Laiyang Central Hospital, Weihai Municipal Hospital, People′s Hospital of Pingliang City

b: Children’s Hospital of Fudan University, Anhui Provincial Children’s Hospital, Haikou People’s Hospital, Sanya Central Hospital

c: Ningbo Women and Children’s Hospital, The Third Affiliated Hospital of Wenzhou Medical University, and Wenzhou Central Hospital of Wenzhou

d: The First Affiliated Hospital of Jinan University, The First People’s Hospital of Tianmen City, The Third Affiliated Hospital of Southern Medical University, The Affiliated Hospital of Xiangnan University
